# Cryo-Electron Microscopy Provides Mechanistic Insights into Solution-Dependent Polymorphism and Cross-Aggregation Phenomena of the Human and Rat Islet Amyloid Polypeptides

**DOI:** 10.1021/acs.biochem.5c00042

**Published:** 2025-05-26

**Authors:** Dylan Valli, Saik Ann Ooi, Ibrahim Kaya, Asger Berg Thomassen, Himanshu Chaudhary, Tobias Weidner, Per E. Andrén, Michał Maj

**Affiliations:** † Department of Chemistry - Ångström Laboratory, 8097 Uppsala University, Lägerhyddsvägen 1, 751 20 Uppsala, Sweden; ‡ Department of Chemistry and Molecular Biology, 3570 University of Gothenburg, Medicinaregatan 7B, 413 90 Gothenburg, Sweden; § Department of Pharmaceutical Biosciences, Spatial Mass Spectrometry, Science for Life Laboratory, Uppsala University, BMC 591, 75124 Uppsala, Sweden; ∥ Department of Chemistry, 1006 Aarhus University, Langelandsgade 140, Aarhus C 8000, Denmark

## Abstract

Inhibitors targeting amyloids formed by the human Islet Amyloid Polypeptide (hIAPP) are promising therapeutic candidates for type 2 diabetes. Peptide formulations derived from the nonamyloidogenic rat IAPP (rIAPP) sequence are currently used as hIAPP mimetics to support insulin therapy. rIAPP itself acts as a peptide inhibitor; yet, the structural-level consequences of such inhibition, particularly its impact on amyloid polymorphism, have not been studied in detail. Here, we conduct coaggregation experiments with varying rIAPP-to-hIAPP concentration ratios and employ high-resolution cryo-electron microscopy (Cryo-EM) to elucidate the polymorphism of the resulting fibril structures. Our results demonstrate that the polymorphism of hIAPP amyloids is highly sensitive to the electrostatic environment, which can be modulated by buffer composition, the concentration of the inhibitor, and cosolvents such as hexafluoroisopropanol (HFIP). Under native conditions, rIAPP associates with hIAPP but does not cross-aggregate, resulting in fibrils primarily composed of hIAPP. Significant inhibition is observed at relatively high concentrations of rIAPP. However, trace amounts of HFIP disrupt this inhibition, leading to increased fibril concentrations due to the formation of cross-seeded products composed of both hIAPP and rIAPP, as evidenced by mass spectrometry and two-dimensional infrared (2D IR) spectroscopy. These findings highlight the critical role of experimental conditions, particularly the electrostatic environment, in modulating amyloid polymorphism, cross-seeding, and inhibition. By providing structural insights into these processes, this study advances our understanding of peptide aggregation and offers valuable guidance for the rational design of more effective therapeutic inhibitors targeting hIAPP-related amyloidosis.

## Graphical abstract

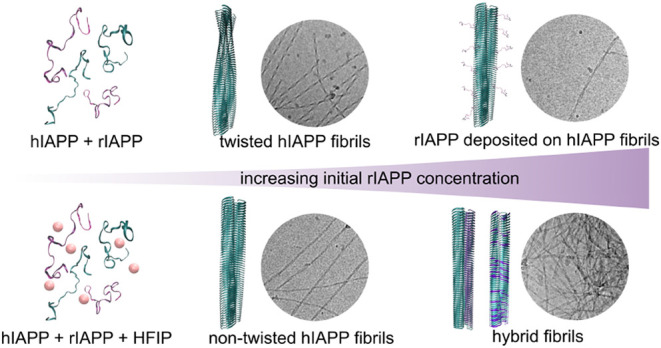

## Introduction

The aggregation of misfolded proteins into amyloid fibrils is associated with a multitude of human diseases.^1^ These aggregation processes are influenced by numerous factors and manifest in many organs throughout the human body.^2^ While there are similarities in fibril structures and aggregation mechanisms, the precise triggering conditions for protein self-assembly remain difficult to establish.^3^ Consequently, significant research efforts are dedicated to developing molecular inhibitors that target not only the final amyloid aggregates but also the intermediates in the aggregation pathway.^4^ In particular, for human Islet Amyloid Polypeptide (hIAPP), inhibiting fibril formation may enhance the survivability of insulin-producing β-cells, an important factor in the pathogenesis of type 2 diabetes.^5^

hIAPP is a 37-amino-acid hormone peptide produced and co-secreted with insulin in the pancreatic islets.^6^ It plays a key role in regulating blood glucose levels, gastric emptying, and signaling satiety.^7^ Despite its physiological importance, hIAPP is also identified as the primary component of amyloid aggregates found in the pancreatic islets.^8^ The aggregation process is highly sequence-dependent, as multiple site-specific mutations significantly reduce the aggregation rate.^9–12^ Rodents serve as important models for understanding how specific amino acid substitutions influence IAPP aggregation.^13^ A key observation is that rat IAPP (rIAPP) does not form amyloid fibrils, and rats seldom develop diabetes.^14,15^ Figure 1 shows the primary sequences of human and rat IAPP. They are identical in the first 17 and last 8 amino acids, but five of the six differences lie between residues 23 and 29. This region—often called the FGAIL region or the amyloidogenic core of IAPP—includes three proline substitutions in the rat sequence that effectively inhibit aggregation.^15,16^ Such a dramatic effect on aggregation propensity, with minimal effect on hormonal activity such as binding to calcitonin receptors, has led to the development of therapeutic agents like Pramlintide, an effective complement to insulin therapy in diabetes treatment.^17^

**Figure 1:**
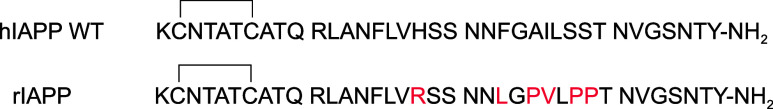
Sequences of WT hIAPP and rIAPP. Residues differing between hIAPP and rIAPP are highlighted in red. The most significant variations occur in and around the amyloidogenic FGAIL region, with key substitutions of proline residues in the rat sequence, which are believed to contribute to its resistance to amyloid fibril formation.

Rat IAPP has been proposed to act as an inhibitor of amyloid formation.^18^ Its inhibitory effect may arise from interfering with the self-assembly process by trapping hIAPP in off-pathway, nontoxic oligomers.^19^ Insulin has also been shown, through coimmunoprecipitation experiments, to bind both monomeric and prefibrillar hIAPP, suggesting it stabilizes hIAPP and hinders aggregation in secretory granules.^20^ These observations highlight the crucial role of peptide-induced inhibition in preventing hIAPP fibril formation within pancreatic islets and emphasize the importance of understanding the structural and molecular details of these processes.

One proposed mechanism of peptide-induced inhibition is a classic β-sheet breaker approach, where the amyloidogenic core of the inhibitor peptide is mutated to prevent self-assembly.^21^ At the same time, another part of the peptide facilitates intermolecular interactions away from the core. In hIAPP, this process is supported by the N-terminal interactions involving residues 1 to 22.^18,22^ Middleton et al. provided residue-specific insights into this mechanism using two-dimensional infrared (2D IR) spectroscopy of isotope labels.^23^ The 2D IR spectra revealed that rIAPP selectively disrupts the N-terminal β-sheet of hIAPP fibrils while the C-terminal region is left intact. Over longer time scales, rIAPP itself adopts β-sheets, likely templated by the exposed regions of hIAPP fibrils. Consequently, rIAPP can both inhibit fibril growth and participate in cross-seeding to form hybrid fibrils, possibly explaining why it is not as effective inhibitor as certain small molecules such as polyphenols.^24^

Numerous studies suggest hIAPP can seed the aggregation of otherwise nonamyloidogenic peptides, including Pramlintide and rIAPP.^23,25–27^ Evidence comes from techniques such as mass spectrometry, Thioflavin-T fluorescence assays, 2D IR spectroscopy, electron microscopy, and atomic force microscopy. Molecular dynamics (MD) simulations propose additional mechanisms for rIAPP and hIAPP coaggregation, but experimental validation remains challenging.^28^ Thus, several crucial aspects of cross-seeding between hIAPP and rIAPP remain unclear, particularly regarding how these interactions lead to the formation of specific amyloid polymorphs.

Understanding amyloid formation requires careful control over the experimental conditions, particularly the initial state of the peptide. Amyloidogenic peptides such as hIAPP are prone to forming pre-existing aggregates or seeds that can affect reproducibility. To address this, hexafluoroisopropanol (HFIP), a fluorinated alcohol, is widely used. HFIP is a polar solvent known to form very strong hydrogen­(H) bonds and is extremely volatile. These properties allow it to effectively disrupt intermolecular hydrophobic interactions and H-bonds within aggregates.^29^ Consequently, dissolving the peptide in neat HFIP followed by evaporation or lyophilization is a standard procedure to disaggregate amyloidogenic peptides and obtain a reproducible, monomeric starting material for aggregation assays and for generating reliable distributions of polymorphs suitable for high-resolution structural analysis.^30^

Cryo-electron microscopy (Cryo-EM) has emerged as the most powerful imaging technique for identifying and quantifying distinct amyloid polymorphs. To date, high-resolution structures of at least six polymorphic forms of hIAPP fibrils have been resolved, each distinguishable by parameters such as crossover length or helical twist.^31–35^ Understanding how environmental factors influence the formation of these polymorphs is of considerable interest. Variations in buffer composition, cosolvents, ionic strength, and the presence of interacting molecules like rIAPP can significantly affect amyloid assembly pathways and resulting fibril structures. Transmission electron microscopy (TEM) studies highlight the role of ionic strength, with different salts potentially inducing different fibril conformations.^36^ However, the magnitude of this effect and the relative populations of specific polymorphs remain to be determined. In particular, even in the absence of salts, our previous cryo-EM study demonstrated that the buffer composition alone can govern fibril morphology during hIAPP fibril formation.^37^ Recent work further emphasizes the importance of the electrostatic environment; for instance, a cryo-EM study of hIAPP fibrils formed in a salt-free environment in the presence of 2% HFIP revealed novel polymorphs (termed SF and TF).^35^ These polymorphs, although structurally distinct in their overall architecture (dimeric vs tetrameric), shared a similar ζ-shaped protofilament structure with a conserved core region (residues 21–29). This contrasts with polymorphs typically observed in standard buffers and highlights the sensitivity of hIAPP polymorphism to solvent electrostatics.

In the present study, we conduct extensive coaggregation experiments with human and rat IAPP to explore the structural outcomes of their interactions. Using Cryo-EM, we characterize the polymorphic fibril structures formed under varying hIAPP-to-rIAPP ratios and in different buffer environments. Our aim is to determine how rIAPP affects the amyloidogenic pathways of hIAPP, thus providing insights into the molecular basis of its inhibitory effect. We also investigate how environmental factors, such as cosolvents, influence amyloid formation, polymorphism, and the extent of cross-aggregation. In addition, we demonstrate that the Thioflavin-T (ThT) binding efficiency depends on fibril polymorphism. Taken together, our results suggest that the modulation of the electrostatic environment is a key determinant governing amyloid polymorphism.

## Results

### Aggregation Kinetics and Inhibitory Action of rIAPP

To investigate the time scales of amyloid fibril formation, we performed Thioflavin-T (ThT) fluorescence assays.^38^ The self-assembly process, as monitored by ThT fluorescence, is divided into three distinct phases: the lag phase, the elongation phase, and the steady phase. These phases are characteristic of the nucleation-driven mechanism of aggregation.^39^ For inhibition purposes, the first two phases are particularly significant. The lag phase signals the onset of amyloid β-sheet formation, while the elongation phase provides insight into the duration of the overall aggregation process. The ThT aggregation kinetics, highlighting the inhibitory action of rIAPP, are presented in Figure 2a. We maintain a constant concentration of hIAPP, while the ratio of hIAPP to rIAPP varies in the range of 1:1 to 1:20. The presence of the aggregation products at the highest concentration of the inhibitor is then tested using cryo-EM screening, which is presented in Figure 2b.

**Figure 2:**
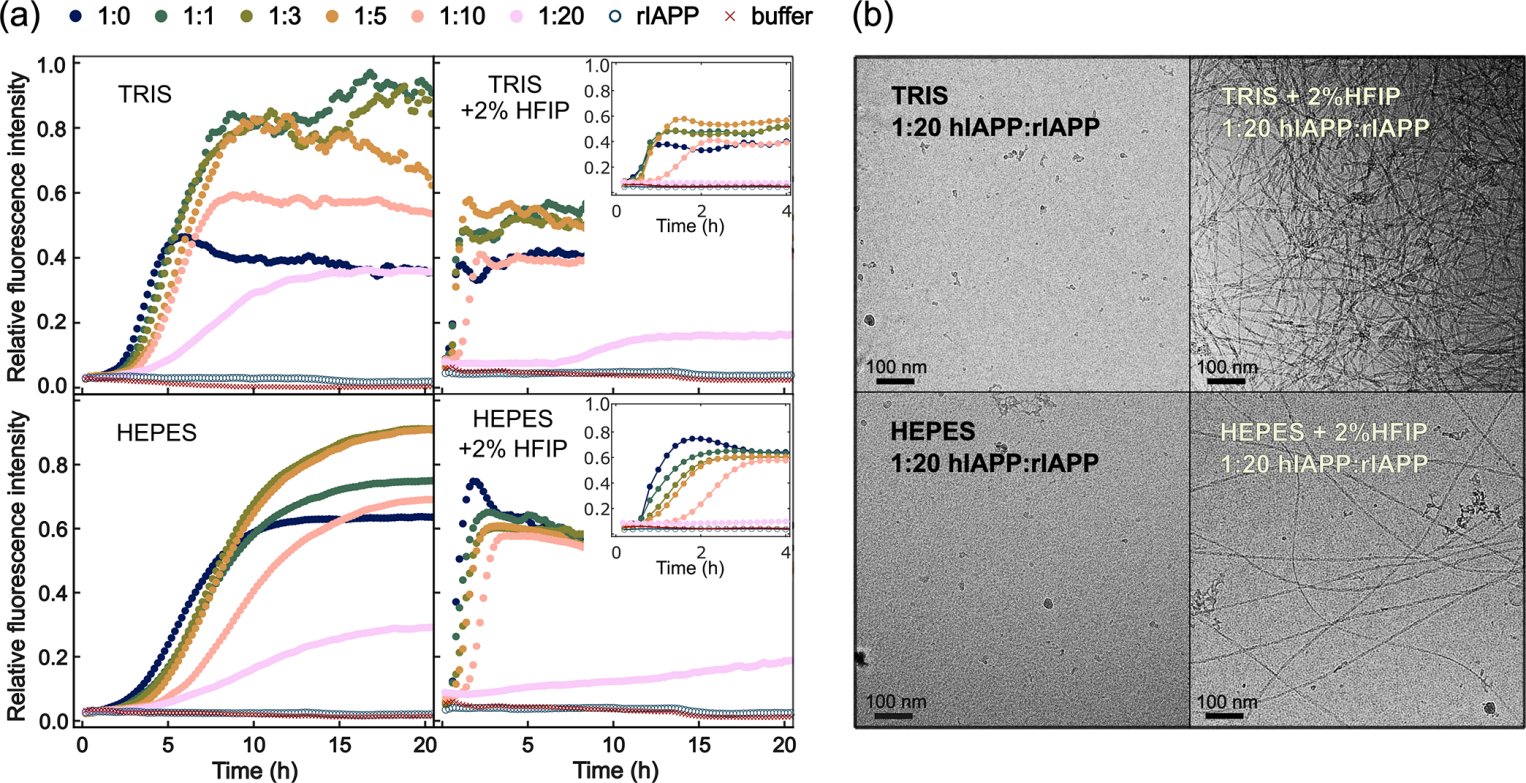
Coaggregation kinetics of hIAPP and rIAPP mixtures in different buffer solutions. The hIAPP concentration is constant across all measurements at 14 μM. The rIAPP concentration ranges between 0 and 280 μM. Ratios of hIAPP to rIAPP are indicated. (a) ThT fluorescence kinetics was performed in Tris buffer 20 mM, HEPES buffer 10 mM, and the same buffers supplemented with 2% HFIP. Fluorescence intensity was normalized to the same value across all data sets to allow direct comparison of ThT intensities between buffer conditions. Increasing the rIAPP concentration extends the lag phase and elongation phase of the aggregation, suggesting inhibition. The addition of 2% HFIP to the aggregation solutions speeds up the aggregation process to the point where inhibition is not observed until the highest concentration of rIAPP. The insets show detailed kinetics of the first 4 h of aggregation. (b) Representative Cryo-EM micrographs of a mixture of hIAPP and rIAPP. No fibrils are observed in the absence of HFIP in the aggregation solution, whereas a high number of fibrils are seen in the presence of HFIP despite extremely low fluorescence intensities in both samples.

An inhibition study conducted in Tris buffer reveals that increasing the concentration of rIAPP impacts amyloid fibril formation. However, despite the extended lag phase and slightly slower aggregation, the final fluorescence intensity is higher in the presence of rIAPP up to a concentration ratio of 1:5. At higher concentrations, inhibition becomes more pronounced, with further increases in the lag and elongation phases and a steady decrease in the fluorescence intensity. At the highest concentration (1:20 ratio), the fluorescence intensity is significantly lower than at other ratios but not much lower than that of the pure wild-type (WT) peptide. To verify whether rIAPP remains ineffective at inhibiting fibril formation even at such high concentrations, we screened the samples at the 1:20 ratio using Cryo-EM. Surprisingly, we detected no fibrils on the majority of grid holes. Although some short fibrils can be found when scanning large areas, most particles are composed of small irregular oligomeric species, which may explain the observed ThT fluorescence intensity even at high concentrations of inhibitor, as it may still be possible for ThT to bind to those oligomers. Nevertheless, based on the micrographs alone, we do not observe any clear differences in the population of oligomeric species across the studied samples.

The addition of 2% HFIP to the Tris buffer dramatically impacts the overall kinetics. Most noteworthy, the lag phase becomes extremely short in the majority of samples, with aggregation starting within the first hour as opposed to 3–4 h in the buffer without HFIP. Some signs of inhibition appear at a 1:10 ratio, with a slight increase in lag phase, but the overall fluorescence intensity is not much affected. Only at a 1:20 ratio, we observe a significant increase in the aggregation times and the fluorescence intensity drops to about 20% of the value observed for the pure WT peptide. This may indicate that the inhibition is stronger at a 1:20 ratio when HFIP is present. In order to confirm this, we carried out cryo-EM screening of the final aggregation products and discovered extremely dense and tangled webs formed by amyloid fibrils (see Figure 2b). This implies that ThT fluorescence intensity is an unreliable measure of fibril concentration in the presence of peptide inhibitors and cosolvents like HFIP.

The ThT aggregation kinetics in HEPES buffer exhibit trends similar to those observed in Tris buffer. Initially, the ThT fluorescence signal increases to a concentration ratio of 1:5 and decreases at higher concentration ratios. The lag phase consistently increases in a concentration-dependent manner. A primary difference in HEPES buffer is that the final ThT intensity at the 1:20 concentration ratio is approximately 20% of the intensity observed for WT hIAPP, indicating a more effective inhibition compared to Tris. Cryo-EM screening at this high ratio detected minuscule amounts of fibril species, suggesting that rIAPP effectively inhibited fibril formation. The residual ThT intensity is likely attributable to oligomeric species and small amounts of fibrils hardly seen under the cryo-EM microscope, similar to observations in the Tris buffer.

As HFIP is added to HEPES, the aggregation kinetics spike rapidly within the first hour. Interestingly, samples containing rIAPP consistently show lower ThT intensities compared to that of the WT peptide. The reduction in ThT intensity is gradual and correlates perfectly with an increasing inhibitor concentration. Up to a concentration ratio of 1:10, a slight increase in the lag phase was observed, but the final fluorescence intensity remains relatively consistent across samples. However, at a 1:20 ratio, aggregation becomes significantly slower and the ThT signal is reduced to about 20% of the WT intensity.

Cryo-EM screening of samples in HEPES with added HFIP revealed the formation of amyloid fibrils, although with a lower concentration of fibrils on the prepared grids in comparison to that in Tris. This suggests that inhibition may be slightly more effective in the HEPES buffer compared to the Tris buffer when HFIP is present.

### Observation of Fibril Morphology with Cryo-EM

To study the potential effects of the aggregation environment and the presence of rIAPP on fibril structures and their distribution, we performed cryo-EM analysis at four different hIAPP:rIAPP concentration ratios. Approximately 1000 micrographs were collected for each condition, which amounts to about 16,000 micrographs across the entire data set. We apply magnification of 73,000×, which results in the pixel size of 1.95 Å/px. Although this pixel size corresponds to a Nyquist limit of roughly 4 Å and, in principle, allows for the generation of high-resolution 3D maps, we found that achieving such resolution in practice is challenging given the relatively limited number of micrographs per condition. In our data sets, the best 3D reconstruction (Figure S1) reached a resolution of about 7 Å. Consequently, we prioritized broader coverage of grid regions to capture the diversity of fibril morphologies rather than attempting to solve high-resolution models for each polymorph. The 2D class averages and polymorph distribution statistics are presented in Figure 3. While several hIAPP structures have been published using different aggregation conditions, only a handful of particles in those studies typically support high-resolution reconstruction, which partly explains why only a limited number of polymorphs have been resolved to atomic detail.^31,32,34^ Our results show a wide range of polymorphs present in the samples, ranging from highly twisted filaments to elongated, flat fibrils. The first and second class averages shown in Figure 3a are particularly similar to the polymorphs identified in recent cryo-EM studies of hIAPP structure, suggesting that these were formed by hIAPP alone, without rIAPP contribution. A preliminary 3D classification of these particles reveals that they all correspond to the common double S-shaped structure previously published (PDB: 7M64).^32^ The projections of the 3D classes are presented in Figure S1 in the Supporting Information. Interestingly, the second class shows that two of these filaments are often found in close proximity, running parallel with a slight offset. While these class averages exhibit higher resolution and reveal internal features, the other class averages appear more blurred, which can be attributed to structural disorder and heterogeneity among the classified particles.

**Figure 3:**
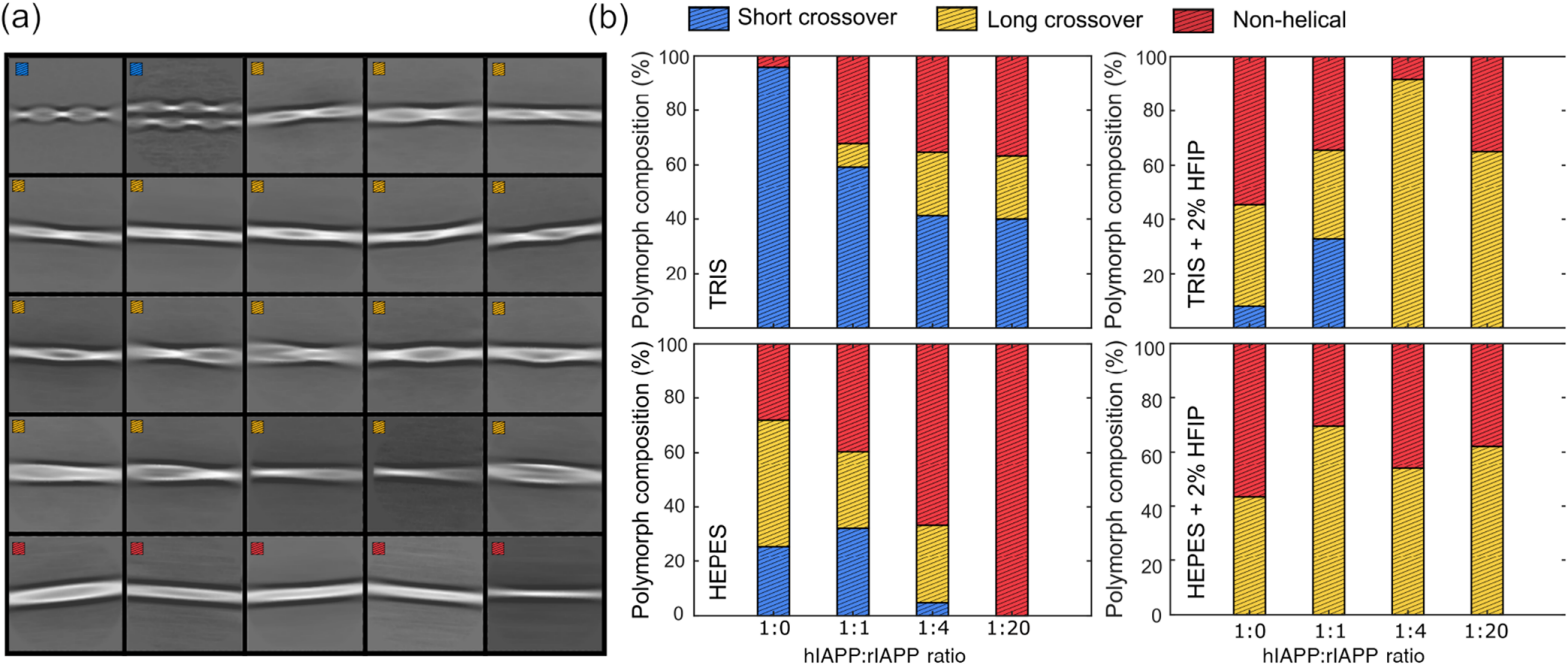
Cryo-EM polymorph distribution of hIAPP aggregated in different conditions. (a) Representative class averages obtained from cryo-EM across all conditions tested. Polymorphs are grouped by the crossover length. A crossover of 260 Å corresponds to the S-shaped structure and is classified as “Short crossover”. Polymorphs with longer crossover are grouped as “Long crossover,” and polymorphs that do not show any twisting feature are grouped as “Non-helical.” (b) Distribution of fibril polymorphs in different aggregation buffers as a function of rIAPP concentration with a fixed hIAPP concentration of 14 μM.

The polymorphic distribution shown in Figure 3b indicates that the aggregation conditions can affect the structural conformation of the peptide. Aggregation in Tris buffer is characterized by a high representation of the double-S polymorph. A trend is observed with increasing rIAPP concentration, where the percentage of highly twisted fibrils decreases, giving way to a higher proportion of elongated and flat fibrils. In Tris buffer, the saturation point is reached rapidly, and the distribution of polymorphs does not appear to differ significantly when comparing the ratios of 1:4 and 1:20.

The addition of HFIP to the Tris buffer results in a substantial decrease in the population of the highly twisted polymorph. Although some of these polymorphs are found at low concentrations of rIAPP, higher concentration ratios yield only long crossover polymorphs and nonhelical structures, without any well-defined trend.

In HEPES buffer, the concentration of the double-S polymorph is significantly lower, and more particles correspond to structures with longer crossover distances. Nevertheless, all helically twisted fibrils decrease in population as the amount of rIAPP increases, and nonhelical fibrils form instead. Interestingly, at a 1:20 ratio, all observed fibrils correspond to flat, nonhelical structures. The results highlight a clear trend of a loss in helicity as a function of rIAPP concentration.

Upon addition of HFIP to the HEPES buffer, the short crossover polymorphs disappear entirely and the trend of losing helicity with increasing rIAPP concentration is no longer observed. It appears that the distribution of long crossover and nonhelical fibrils is approximately equal and is independent of the concentration of the peptide inhibitor.

To assess reproducibility, we performed independent replicate experiments for WT hIAPP in TRIS, WT hIAPP in HEPES, and the TRIS sample containing hIAPP and rIAPP at a 1:20 ratio with 2% HFIP. In each case, the polymorphic distribution was consistent with the results presented in Figure 3b. As an example, a replicate data set for WT hIAPP in HEPES revealed the following polymorph populations: 35% short crossover, 35% long crossover, and 30% nonhelical fibrils. This distribution closely mirrors the main data set, differing by only 5% in the relative abundance of the short crossover polymorph. Similarly, for WT hIAPP in TRIS, we observed the dominant presence of short crossover fibrils in all replicates. While only the highest quality data sets were used for structural analysis, these findings suggest reliable reproducibility across independent measurements.

It is also worth mentioning how the inhibition process is reflected in the number of particles picked in the cryo-EM data. The relative number of particles measured over the same area of the grid is presented in Figure 4. We recognize that particle distributions on grids can naturally exhibit some variability, arising from the vitrification process and sample heterogeneity. Furthermore, like any image-based quantification, the selection of areas for analysis and inherent randomness in particle settling can influence the absolute counts. However, by consistently applying this quantification across comparable micrograph regions for each sample, the method still offers a valuable qualitative assessment of relative fibril abundance under the different conditions.

**Figure 4:**
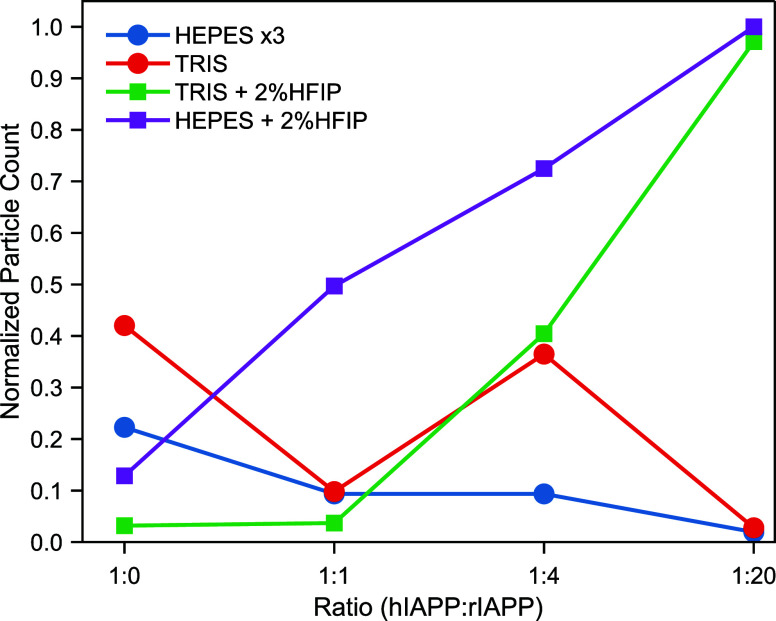
Quantification of helical segments extracted in RELION across aggregation conditions. Normalized number of helical segments extracted from 1000 cryo-EM micrographs for each condition. Counts were normalized relative to a total of 4.3 million extracted particles. The interbox distance was kept at 3 asymmetrical units and a helical rise of 4.75 Å. For visualization purposes, the particle count for the HEPES condition was multiplied by three due to its lower relative abundance compared to that of other conditions.

In the absence of HFIP, increasing the rIAPP:hIAPP ratio generally resulted in a lower normalized particle count, consistent with an inhibitory effect of rIAPP, especially evident at the 1:20 ratio, where particle counts were very small. However, this trend is reversed in the presence of HFIP, where the number of particles increased with higher concentrations of rIAPP, reaching a maximum at the 1:20 ratio. These opposing trends, where a higher concentration of inhibitor results in more fibrils, suggest that HFIP promotes aggregation or facilitates cross-seeding, leading to the formation of hybrid hIAPP:rIAPP fibrils. To gain insight into the possibility of cross-seeding, we applied mass spectrometry and 2D IR spectroscopy for a detailed analysis of the fibril composition.

### MALDI-FTICR-MS and 2D IR Analysis of Fibril Composition

To estimate the relative content of hIAPP and rIAPP within the fibrils formed under various solution conditions, we employed matrix-assisted laser desorption/ionization (MALDI) Fourier transform ion cyclotron resonance (FTICR) mass spectrometry. To minimize potential bias arising from nonuniform sample deposition or preferential crystallization within the matrix, we applied the MALDI mass spectrometry imaging (MALDI-MSI) method. Spectra were acquired across the entire area of the dried sample spot on the MALDI target. An example of the measured mass spectra is shown in Figure 5a. The spectrum displays two prominent peaks corresponding to hIAPP and rIAPP, with *m*/*z* values of 3903.8 and 3920.8, respectively, matching their expected molecular weights. By analyzing the relative peak intensities, we estimated the relative abundance of each peptide within the fibril samples. This approach does not require an internal standard since the analysis relies explicitly on determining the ratio of the two components rather than their absolute quantities. Such a relative measurement, averaged over the entire sample deposit via MSI, mitigates concerns related to localized crystallization or extraction differences. Additionally, the structural similarity between the peptides supports the assumption of comparable ionization efficiencies.

**Figure 5:**
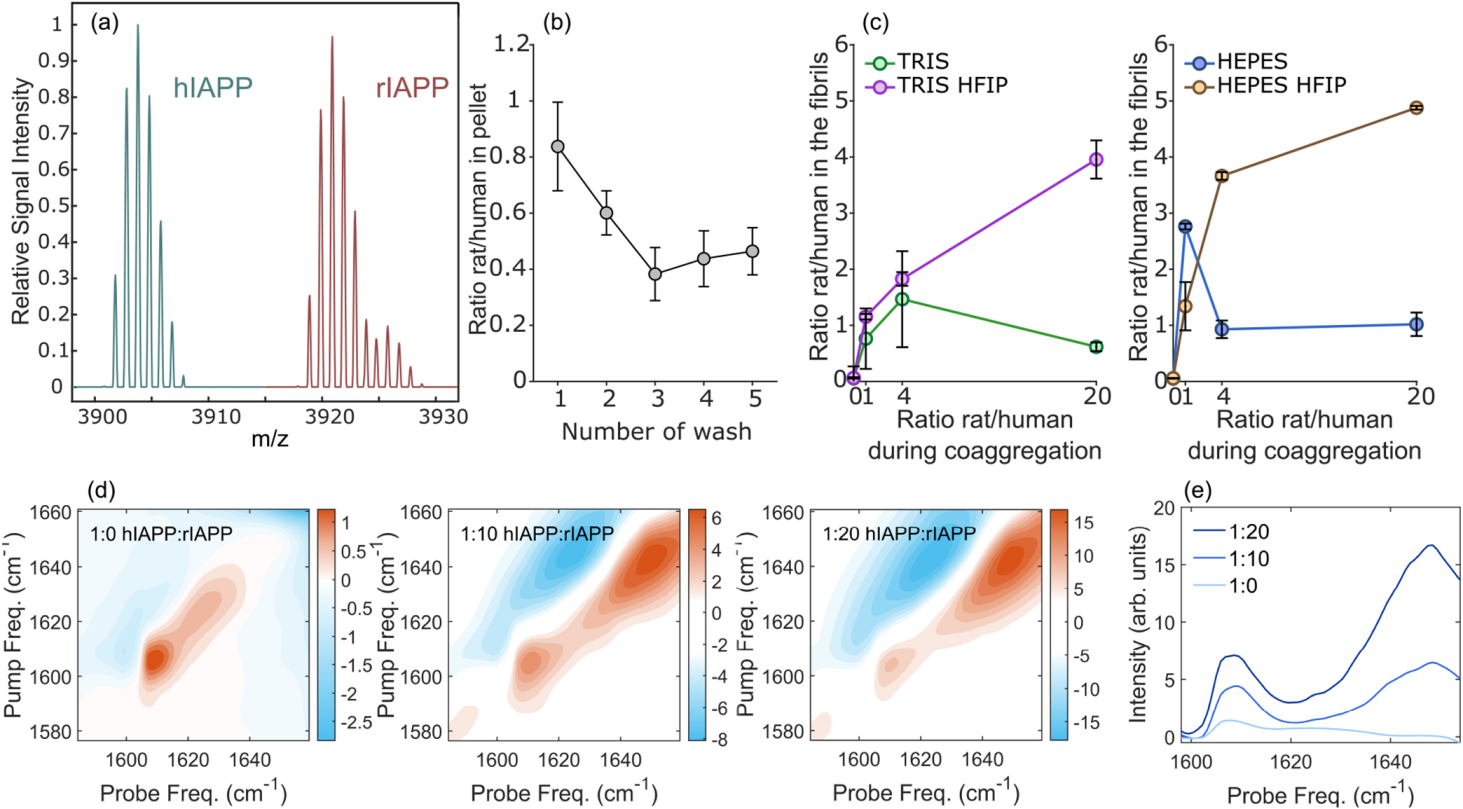
MALDI-FTICR-MS analysis of the peptides present in fibrils formed under different conditions. (a) Mass spectra of a 1:1 mixture of hIAPP and rIAPP in Tris buffer. The main peaks correspond to the expected masses at *m*/*z* 3903.8 (teal) and 3920.8 (red) for hIAPP and rIAPP, respectively. (b) Ratio of rIAPP to hIAPP measured by MALDI-FTICR-MS from peak areas in the same sample as in (a) as a function of the number of wash cycles. The detected rIAPP decreases after the first three cycles and then stabilizes, indicating the removal of peptides coating the fibrils. (c) Ratios of rIAPP to hIAPP measured by MALDI-FTICR-MS in different aggregation solutions as a function of increasing rIAPP concentration. (d) 2D IR spectra of fibrils formed in Tris +2% HFIP at three different hIAPP:rIAPP ratios: 1:0 (left), 1:10 (middle), and 1:20 (right). The strong feature near 1610–1620 cm^–1^ indicates β-sheet structure, while the peak near 1650 cm^–1^ corresponds to unaggregated peptide. (e) Overlaid diagonal slices (blue lines in each panel) highlight how the relative intensity of the amyloid versus monomeric signals evolves with increasing rIAPP content.

First, it was crucial to determine whether the detected rIAPP signals originated from rIAPP incorporated into the fibrils or from monomeric rIAPP molecules nonspecifically associated with the fibril surfaces. To address this, we performed multiple cycles of washing and pelleting the fibrils via centrifugation. After each cycle, the fibrils were deposited onto the MALDI MTP ground steel target and mass spectra were acquired. As shown in Figure 5b, the relative rIAPP signal decreased significantly during the initial washes, approximately halving within the first three cycles before reaching a plateau. This observation suggests the removal of loosely or nonspecifically bound peptides. Although the signal stabilized after three washes, we performed five wash cycles on all subsequent samples as a standard protocol to ensure thorough removal of nonincorporated material. To further probe if the rIAPP signal at the plateau level represented fibrillar or still surface-associated rIAPP, we employed additional washes using sarkosyl, which is known to disrupt weaker, nonspecific protein–protein interactions. These sarkosyl washing experiments confirmed that for fibrils formed in the absence of HFIP, a substantial fraction of the detected rIAPP was indeed surface-associated and was consequently removed by the surfactant treatment (see Figure S2 for details).

The rIAPP incorporation ratios for fibrils formed under different aggregation conditions are listed in Figure 5c.

In Tris buffer without HFIP, increasing the rIAPP concentration results in only minor changes in rIAPP incorporation into the fibrils. There appears to be a small increase up to the ratio of 1:4, and the incorporation measured at 1:20 is very similar to that at 1:4, suggesting a plateau over this range. On the other hand, adding 2% HFIP to the Tris buffer leads to a significant increase in the rIAPP ratio, suggesting that HFIP promotes the incorporation of rIAPP into the fibrils through cross-seeding. At the highest studied concentration, 3 rIAPP molecules were incorporated into the fibrils per 1 hIAPP molecule.

A similar trend was observed in the HEPES buffer. Without HFIP, rIAPP incorporation was generally low across most tested ratios, although at a 1:1 ratio, there was a slight increase in rIAPP content. However, the addition of 2% HFIP resulted in a marked increase in the rIAPP content within the fibrils, reaching up to 3 rIAPP molecules per hIAPP at higher rIAPP concentrations. These findings provide strong evidence that HFIP facilitates the cross-seeding of hIAPP and rIAPP and promotes the formation of hybrid fibrils composed of both peptides. We assume that the formation of hybrid fibrils is favored over pure rIAPP fibrils due to stability factors and the drive toward minimizing steric clashes across β-strands.

To further confirm the incorporation of rIAPP into the fibrils formed in Tris-HFIP, we applied two-dimensional infrared (2D IR) spectroscopy. Importantly, 2D IR has the advantage that the observed Amide signals originate directly from the protein backbone, eliminating the need to rely on external dyes, as in fluorescence-based assays. The 2D IR spectra are presented in Figure 5d, and the diagonal slices of these spectra are shown in Figure 5e. A detailed discussion of 2D IR spectroscopy and its application to proteins has been provided elsewhere.^40,41^ Briefly, typical amyloid features are apparent at 1610–1625 cm^–1^, consistent with a β-sheet structure, while a peak at 1650 cm^–1^ indicates monomeric/random coil conformations. Although 2D IR spectra include both positive (0–1) and negative (1–2) transitions, analyzing diagonal slices provides an effective FTIR-like view; however, one must note that intensities do not scale identically with those in linear IR spectra.

Pure hIAPP fibrils show only a weak monomeric feature at 1650 cm^–1^, indicating that most hIAPP monomers have aggregated. The main β-sheet peak at 1612 cm^–1^ is prominent. Upon a 10-fold increase in rIAPP concentration, the random coil peak at 1650 cm^–1^ becomes more pronounced, reflecting unaggregated rIAPP. At the same time, the fibril-associated peak at 1612 cm^–1^ intensifies 3-fold, indicating an overall increase in the fibril content. At the highest 1:20 hIAPP:rIAPP ratio, the monomeric feature dominates, yet the β-sheet peak is further enhanced to about five times that of the pure hIAPP sample. These findings are consistent with both cryo-EM particle counts and MALDI-MSI results, proving that rIAPP is indeed incorporated into the fibrils and supporting the reliability of our mass-spectrometric analysis of the cross-aggregated products.

## Discussion

In this study, we investigated the inhibition of hIAPP aggregation by rIAPP and explored its polymorphism and cross-aggregation efficiency, focusing on how environmental conditions influence these processes. By combining ThT fluorescence kinetics, cryo-EM, and mass spectrometry analyses, we gained insights into the inhibitory mechanism of rIAPP on hIAPP aggregation and identified the conditions under which this inhibition is modulated or completely suppressed.

### Mechanisms Behind Amyloid Polymorphism

Our study provides compelling insights into the polymorphism of hIAPP fibrils being primarily controlled by the electrostatics of its environment. Specifically, we demonstrate how the solvent environment modulated by buffer composition and the presence of HFIP, as well as interactions with rIAPP, influences hIAPP aggregation kinetics and fibril polymorphism. The mechanisms highlighted in Figure 6 summarize our findings and propose how electrostatic factors dictate the aggregation pathways of hIAPP.

**Figure 6:**
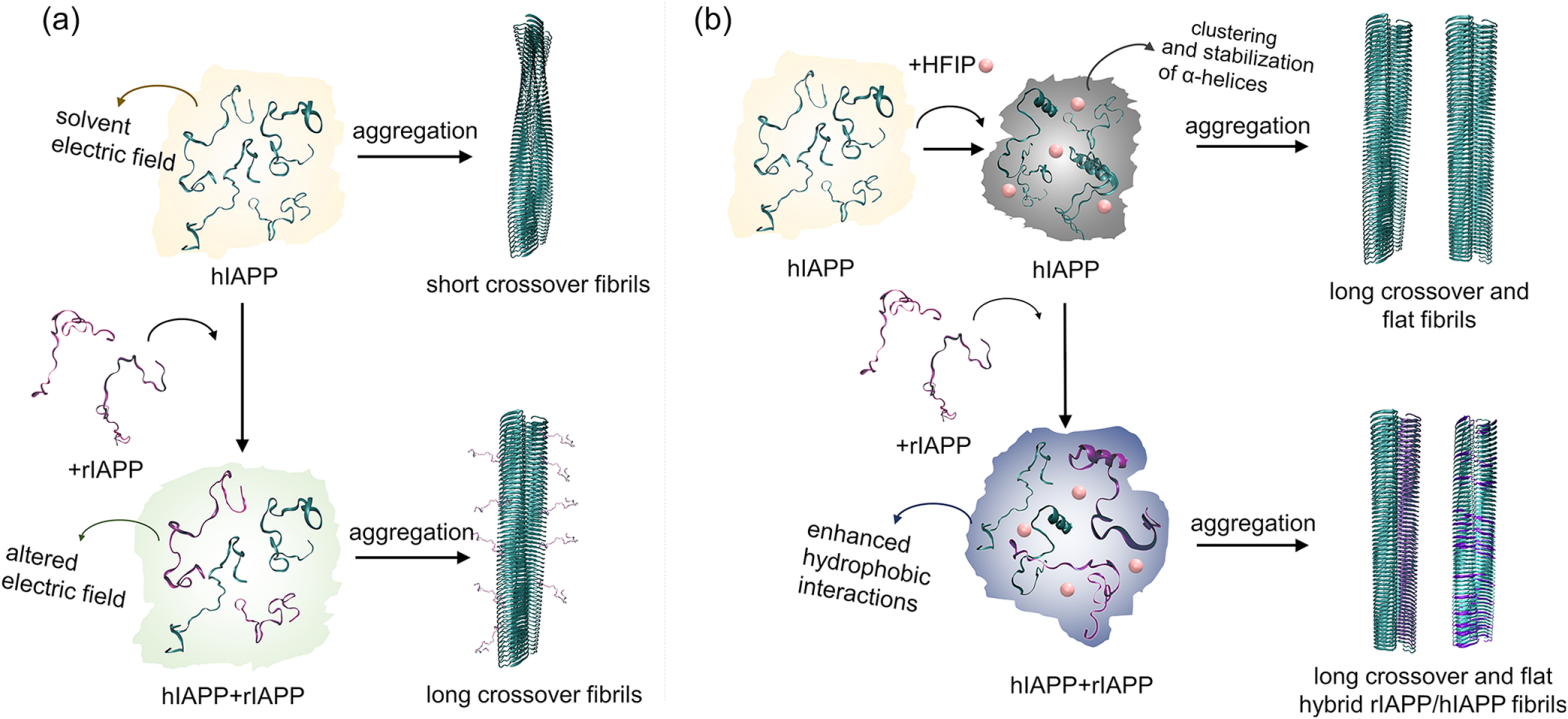
Two mechanistic pathways highlighting the effects of electrostatics on the polymorphism of IAPP fibrils. (a) Schematic showing hIAPP aggregation and inhibition in buffer media without HFIP. The choice of pH and buffer determines the solvation structure and the electrostatic interactions between water and dissolved peptides. The distribution of polymorphs depends on the characteristics of the solvent field and, in most studied cases, results in a large population of helically twisted fibrils. The introduced rIAPP inhibitor interacts with dissolved hIAPP, but its presence also has a general effect on the solvation structure, resulting in the formation of different polymorphs compared with plain buffer. In this scenario, the majority of rIAPP molecules associate on the surface of fibrils formed by hIAPP. (b) Schematic showing the effect of HFIP on the same aggregation processes presented in (a). The addition of HFIP has a profound effect on the local solvation structure via clustering effect, direct interaction with the peptide, or by increasing the lifetime of α-helices at the N-terminus. This effect results in faster aggregation and formation of flat and weakly twisted fibrils. The environment also enhances hydrophobic interactions between rIAPP and hIAPP, in which case the two peptides participate in cross-seeding, and both rIAPP and hIAPP form fibrils.

Electrostatic interactions are fundamental to protein folding and aggregation, as they govern the forces between charged residues and influence peptide conformation and stability.^42^ The ionization state of amino acid side chains is determined by the pH and ionic strength of the solvent, which in turn affects the overall charge distribution of peptides.^43^ In our experiments, the use of different buffers (Tris vs HEPES) and the addition of HFIP created distinct electrostatic environments that significantly impacted hIAPP aggregation behavior. In native buffer conditions (without HFIP), Cryo-EM revealed that hIAPP aggregates into the double S-polymorph which is characterized by extremely short crossover lengths.

The introduction of nonamyloidogenic rIAPP modified the local electrostatic environment allowing hIAPP to sense the presence of the inhibitor and directly interact with it, which also influences the distribution of fibril polymorphs. Previous site-specific mutations and 2D IR studies have shown that the interaction occurs through the N-terminus of the peptide.^18,23^ Contrary to previous studies, our results indicate that rIAPP does not incorporate into hIAPP fibrils under the studied conditions but associates with the fibrillar surface, as demonstrated by our experiments involving a surfactant (Figure S2). rIAPP may compete with hIAPP monomers for binding sites or interact with hIAPP oligomers, forming nonproductive complexes that are off-pathway for fibril formation. This interaction is likely driven by intermolecular interactions at the N-terminus, but structural incompatibilities (due to proline residues in rIAPP) prevent the formation of stable heterogeneous β-sheets.

HFIP is known to influence protein secondary structures by altering H-bonds and promoting hydrophobic interactions. Moreover, at higher HFIP concentrations, due to the change in solvent’s dielectric constant, HFIP reduces electrostatic interactions between charged residues.^44^ A mechanistic perspective is offered by the phase-partitioning model for IAPP fibrillogenesis proposed by Padrick and Miranker,^45^ where a dispersed or off-pathway phase continuously releases low concentrations of soluble peptide that drives nucleation and subsequent fibril elongation. The presence of HFIP could, in principle, accelerate or bypass certain steps in this partitioning model by shifting the solubility equilibrium and potentially raising the concentration of soluble hIAPP monomers available for nucleation. Alternatively, HFIP may contribute to stabilizing structural intermediates, including β-sheet structures that are essential for fibril formation. The formation of small globular clusters has been observed for Amyloid β peptide in 2% HFIP solution.^46^ These clusters disassemble almost immediately after HFIP is removed. It remains possible that early micellar or globular clusters form transiently in our experiments, although we did not directly detect a “dispersed” phase in our system since we examined only the final products of the aggregation process.

Another important effect of fluorinated cosolvents is their ability to stabilize α-helical secondary structures in proteins.^47^ The monomeric state of hIAPP is predominantly a random coil, but transient helical structures at the N-terminus have been detected with nuclear magnetic resonance (NMR) and two-dimensional infrared (2D IR) spectroscopy of isotope labels.^48,49^ These transient α-helices have been hypothesized to influence the aggregation of hIAPP into amyloid fibrils, though their specific role has been the subject of debate. Some studies suggest that helical intermediates act as detrimental species in solution by accelerating aggregation.^50^ In contrast, other research studies indicate that these helical structures are off-pathway, showing that helices do not significantly influence aggregation, and that preventing the formation of N-terminal helices increases cytotoxicity.^51^

Regardless of their precise role, the concentration-dependent circular dichroism (CD) spectroscopy indicates that the α-helical content increases with increasing HFIP concentration.^44^ It is thus possible that the altered distribution of secondary structures at the N-terminus of the studied peptides affects the aggregation outcomes.

In this work, the inclusion of 2% HFIP in the aggregation solutions substantially altered both the kinetics of the hIAPP assembly and the morphology of the resulting fibrils. A critical question is whether the phenomena documented at elevated HFIP levels apply to the relatively low concentration (2%) employed here, particularly in the context of clustering effects highlighted by the partitioning model and the role of transiently formed α-helical structures. Interestingly, even 0.17% HFIP has been reported to exert marked effects on the structure and stability of prion protein (PrP),^52^ suggesting that direct interactions between HFIP and the peptide, rather than bulk changes in the solvent, are primarily responsible. Indeed, concentration-dependent scattering experiments of HFIP-water mixtures have demonstrated extensive HFIP clustering.^53^ Although the maximal scattering occurs at approximately 30% HFIP, a noticeable increase in scattering is observed even at very low HFIP levels, which also correlates well with protein conformational changes.^53^ These findings suggest that amyloid-forming peptides may experience high sensitivity to HFIP-induced microenvironments across a broad concentration range. The stabilization of α-helical structures is more pronounced at high HFIP concentrations, but 2D IR, NMR, and MD simulations reveal that hIAPP forms transient α-helices under native conditions.^48,49,54^ It is therefore possible that even low concentrations of HFIP prolong the lifetime of these transient helices at the N-terminus, which remains to be confirmed experimentally. In the presence of HFIP, rIAPP was found to coaggregate with hIAPP, forming hybrid fibrils, which is shown in Figure 6b. Our mass spectrometry analyses (Figure 5c) provide quantitative insight into this phenomenon, demonstrating that under conditions of high rIAPP excess, the resulting fibrils can incorporate significantly more rIAPP than hIAPP, with final stoichiometries reaching up to 4:1. Although Figure 6b qualitatively depicts the cross-seeding mechanism enabled by HFIP, it does not explicitly detail this stoichiometric imbalance. The observation of rIAPP dominance in the hybrid fibrils suggests that the HFIP-modulated environment may kinetically or thermodynamically favor rIAPP incorporation into the growing fibril structure, especially when it is the vastly more abundant species in solution. Factors such as altered electrostatic/hydrophobic interactions, potentially stabilized aggregation-prone intermediates, or different structural nature of polymorphs formed in HFIP could contribute to a lower barrier for rIAPP addition onto a hybrid fibril end compared to hIAPP.

Our cross-seeding experiments provide additional evidence for this mechanism. Specifically, we observed that mature hIAPP fibrils did not seed rIAPP aggregation in the presence or absence of HFIP (Figure S3). This observation may suggest that the nucleation and cross-seeding are driven by smaller assemblies stabilized by HFIP, which is consistent with observations for Aβ peptide^46^ as well as the model of rIAPP-hIAPP complex derived from 2D IR spectroscopy.^23^ By reduction of electrostatic barriers, HFIP enables rIAPP to integrate into the fibril core, activating cross-seeding despite the structural differences. Furthermore, the increased population of the transient α-helices in HFIP-containing solutions may help stabilize the specific conformations of hIAPP and rIAPP required for coaggregation. The suppressed inhibition due to the presence of HFIP has also been observed for small peptide inhibitors, but the effect was significant only in the presence of phospholipid membranes.^55^ Here, we demonstrate that the function of peptide inhibitors can also become impaired in nonmembrane environments.

### Insights from ThT Fluorescence Assays

The kinetic results obtained from the ThT fluorescence assay present conflicting interpretations. While the assay effectively captures relative changes in the onset of aggregation by showing negligible fluorescence during the lag phase, the final fluorescence intensity does not correlate well with the overall concentration of fibrillar species in the samples. In particular, at a 1:20 hIAPP ratio in Tris buffer, we observed high fluorescence readings, despite nearly empty grids in cryo-EM images. On the other hand, the addition of HFIP resulted in lower fluorescence readings but revealed an extremely high fibril content in the cryo-EM micrographs.

It is well-established that the binding of ThT to amyloid β-sheets depends on multiple factors, including the arrangement of β-sheets and side-chain channels involving aromatic residues.^56^ Aromatic residues are hypothesized to contribute significantly to the hydrophobic surfaces that facilitate ThT binding.^57^ Available cryo-EM structures of hIAPP reveal different orientations and local environments of aromatic residues within the fibril core, suggesting that ThT binding may vary among different polymorphs. A particularly notable case is that of pufferfish IAPP, which forms amyloid fibrils that do not bind ThT at all.^58^ Although the polymorphism of pufferfish fibrils has not been characterized, this observation indicates that certain fibril structures are highly resistant to ThT binding.

Our analysis of ThT data in Tris and HEPES buffers, coupled with the distribution of different polymorphs, provides insights into why fluorescence intensity increases upon the addition of rIAPP, even though cross-seeding and hybrid fibril formation do not occur. We attribute this trend to the formation of higher concentrations of nonhelical polymorphs. This suggests that ThT binds more effectively to flat, nonhelical amyloids than to those exhibiting strong helical twists. Producing larger amounts of flat fibrils would require us to increase the rIAPP concentration ratio further, which could then allow rIAPP to start inhibiting the aggregation process and significantly affect the overall fibril concentration. This would complicate the correlation between fluorescence intensity and fibril concentration without a scaling factor that accounts for the total concentration of fibrillar species. Developing an experimental approach that accurately correlates the distribution of polymorphs, fluorescence intensities, and total concentrations across a broad range of rIAPP ratios would provide a more quantitative and statistically relevant understanding of the observed variations in fluorescence signals during hIAPP aggregation.

Furthermore, it is important to note that ThT binding is influenced by environmental factors such as pH and ionic strength.^59,60^ Therefore, relying solely on quantitative analyses of ThT assays is challenging, and more accurate methods may be required to study inhibition mechanisms. One potential approach is to apply 2D IR spectroscopy to monitor the aggregation kinetics in the presence of inhibitors.^61^A recent 2D IR study demonstrated the capacity to track the formation of two distinct polymorphic forms of IAPP in real time.^62^ Correlating cryo-EM-resolved polymorph distributions with the unique spectral signatures identified by 2D IR would provide a powerful means of directly linking fibril morphology to spectroscopic signals. Such a combined framework would clarify not only how specific fibril architectures evolve but also how they are being affected by the presence of salts, inhibitors, and cosolvents.

## Conclusions

The study demonstrates that the electrostatic environment is a key determinant of hIAPP aggregation kinetics and fibril polymorphism. By modulating electrostatic interactions through buffer composition and the addition of HFIP, we can influence the aggregation pathway of hIAPP and its interaction with peptide inhibitors. The formation of distinct fibril polymorphs and cross-aggregates emphasizes the importance of environmental factors in protein aggregation processes.

Our findings have significant implications for designing therapeutic inhibitors that target hIAPP aggregation. The strong dependence of inhibition by rIAPP on its surrounding environment underscores the need to carefully consider solvation effects and peptide–peptide interactions. Although cosolvents such as HFIP typically do not appear in final drug formulations, the physiological environment is far more complex than a simple buffer system, making it challenging to predict *in vitro* whether cross-aggregation or hybrid fibril formation might occur *in vivo*. Indeed, there is evidence that injecting various amyloidogenic peptides into mice can seed and accelerate fibril accumulation in their pancreatic islets,^63^ emphasizing the potential risks associated with peptide-based interventions. Strategies to enhance the efficacy of peptide inhibitors could involve stabilizing their inhibitory conformations, perhaps through cyclization or incorporation of non-natural amino acids to reinforce β-sheet-disrupting features.^64–66^ Additionally, designing inhibitors that target specific aggregation-prone regions of hIAPP without facilitating cross-seeding could mitigate the risk of hybrid fibril formation. Our study also highlights the importance of experimental design in amyloid research. The use of cosolvents like HFIP, while beneficial for solubilizing peptides, can introduce artifacts or unintended interactions that obscure the true inhibitory mechanisms. Careful control and reporting of experimental conditions are essential for reproducibility and accurate interpretation of the results.

## Materials and Methods

### Peptide Synthesis and Purification

The hIAPP and rIAPP were synthesized on a 0.1 mmol scale using 9-fluorenylmethyloxy-carbonyl (Fmoc) chemistry with a Biotage Initiator+ Alstra peptide synthesizer. The C-terminal amide was formed using Tentagel R RAM resin (0.18 mmol/g). Pseudoproline derivatives were used to prevent aggregation and improve coupling during the synthesis.^67^ For hIAPP, Fmoc-Ala-Thr­(Psi­(Me,Me)­pro)–OH was used at positions 8 and 9, and Fmoc-Leu-Ser­(Psi­(Me,Me)­pro)–OH was used at positions 27 and 28. For rIAPP, only Fmoc-Ala-Thr­(Psi­(Me,Me)­pro)–OH was used at positions 8 and 9. A standard trifluoroacetic acid (TFA)-based cleavage cocktail (95% TFA, 2.5% triisopropylsilane, and 2.5% H2O) was used to cleave the synthesized peptides. Crude peptides were dissolved in H2O and lyophilized. To form the disulfide bridge between Cys2 and Cys7, the peptides were dissolved in DMSO mixed with 25% acetic acid solution at a 60:40 ratio for 24 h. Peptides were then purified using reversed-phase HPLC (Isera C18 preparative column, 250 mm × 20 mm), with a gradient elution composed of buffer A (100% H2O with 0.045% HCl) and buffer B (80% acetonitrile with 0.045% HCl). The eluted peptides were collected and lyophilized overnight. Second purification was carried out by dissolving the peptide in 50% hexafluoroisopropyl alcohol (HFIP) and 20% acetic acid. The eluted peptides were lyophilized overnight. Liquid chromatography–mass spectrometry (LC-MS) confirmed the mass of the synthesized peptides. The purified peptides were dissolved in HFIP for several hours. A 10 μL of peptide was evaporated with nitrogen gas and resuspended in H2O to determine the concentration using absorbance at A280 nm using the extinction coefficient of tyrosine (1490 M^–1^ cm^–1^). The peptides were aliquoted based on the concentration needed and were lyophilized overnight and stored at −20 °C until use.

### Thioflavin-T Fluorescence Assay

IAPP aggregation was monitored by measuring the fluorescence intensity of ThT over 1 week. All samples were prepared in triplicate and measured in a 96-well plate sealed with a black TopSeal-A using a Victor X4 from PerkinElmer. The samples with different ratios of rat to human IAPP were provided with 20 μM of ThT, 14 μM of hIAPP, and a range of rIAPP concentrations from 0 to 280 μM. Measurements were recorded every 10–12 min using an excitation of 450 nm and emission of 490 nm and monitored with the PerkinElmer 2023 Workstation software.

### Cryo-Electron Microscopy

The samples for Cryo-EM were prepared by resuspending the lyophilized hIAPP and rIAPP aliquots in Tris or HEPES buffer (pH 7.4) to make stock solutions. The coaggregations at different concentrations were started immediately by mixing hIAPP and rIAPP from the corresponding stock solutions, and HFIP was added to a final concentration of 2%. All samples were prepared at a hIAPP concentration of 14 μM, and a rIAPP concentration of 0, 14, 56, and 280 μM. The samples were aggregated for 7 days at room temperature without mixing. Quantifoil 3.5/1 grids were glow-discharged for 90 s at 20 mA and 3 μL of sample was applied before plunge-freezing in liquid ethane with a Vitrobot mark IV from Thermo Scientific. For each data set, 1000 micrographs were collected at the Umeå Centre for electron microscopy on a 200 kV Glacios cryo-TEM with a Falcon 4 detector. The micrographs were collected within a defocus range of −2 to −4 μm at a nominal magnification of 73,000× to cover a wide field of view with a pixel size of 1.95 Å/px, and 20 frames were recorded for a total dose of 23 e/Å2. The collection for all data sets was set up using the EPU Multigrid Software. All the following data processing was performed on the Tetralith cluster of the Sweden’s National Super Computer Centre (NSC) in the same way for all data sets unless explicitly mentioned. Preprocessing of the micrographs was done within RELION 4.0 with the built-in Motion Correction algorithm, followed by CTF estimation using CTFFIND-4.1. The 3dem implementation of Topaz for fibrils was used to train an autopicking model based on 100 manual picked micrographs, and all fibrils start-end coordinates were automatically picked. The particles were extracted with a box size of 512 pixels and an interbox distance of 3 asymmetrical units of 4.75 Å. For the data sets containing a large number of particles, the extraction box was down-sampled twice due to memory issues. A first 2D classification was run with 200 classes and a T value of 2 using the EM algorithm and ignoring CTFs until the first peak. All classes containing fibrils were then selected and used for a second round of 2D classifications with a T value of 4. Analysis of the polymorphic distribution was performed based on the 2D classifications by visually comparing the class averages and measuring the crossover distances with ImageJ. For classes that were too blurry or for those where the identification of the polymorph was unclear, particles were re-extracted with a 256 px box and subjected to a similar 2D classification process. The total numbers of picked particles and selected particles, after removing nonfibrillar segments due to contamination or very low-quality images, are reported in Table S1.

### MALDI-FTICR-MS Analysis

All of the samples were diluted 2-fold prior to MALDI-MS analysis in order to place the intensity values for selected peptide ions in a linear range. MTP 384 ground steel target plates (Bruker Daltonics) spotted with 0.5 μL of extracts were sprayed with 2,5-dihydroxybenzoic acid (35 mg/mL in 50% ACN and 0.2% TFA) matrix in six passes at 75 °C using an automatic TM-sprayer (HTX-Technologies LLC, Chapel Hill, NC) with the following parameters: nitrogen pressure 6 psi, solvent flow rate of 70 μL/min, nozzle head velocity of 110 cm/min and 2 mm track spacing. The analysis was performed on a MALDI Fourier transform ion cyclotron resonance (FTICR) mass spectrometer (solariX 7T-2ω, Bruker Daltonics, Bremen, Germany) equipped with a Smartbeam II 2 kHz laser and operated in positive-ion mode. All data were acquired in continuous accumulation of selected ion (CASI) mode with a 50-Da mass window in the mass range 300–5000 *m*/*z* (CASI 3865–3965 *m*/*z*). The operating conditions (ion transfer, laser power, and laser focus) were adjusted to maximize the signal of the relevant parent ions before the first data acquisition and then kept constant throughout the analysis of the complete experiment. The method was calibrated externally with red phosphorus over an appropriate mass range. The experiment was set up in imaging mode at 250 μm lateral resolution, firing 100 laser shots per pixel. The peak area of [M + H]+ hIAPP and rIAPP peptide ions per pixel per spot is used for the data analysis. The ratios of the peak areas of rIAPP to hIAPP were calculated in different conditions.

### Two-Dimensional Infrared (2D IR) Spectroscopy

Two-dimensional infrared (2D IR) spectra were collected by using a 10 kHz time-domain 2D IR spectrometer (2DQuickIR, PhaseTech). A Yb-based Pharos laser (Light Conversion) delivered 100 fs pulses centered at 1030 nm. Broad mid-IR pulses spanning the Amide band region were generated by using an ORPHEUS optical parametric amplifier and a Lyra difference-frequency mixing module (Light Conversion). The resulting mid-IR beam was split into pump and probe pulses by using a beam splitter. The pump was directed to a Ge-based acousto-optic modulator pulse shaper (PhaseTech) to generate a pair of time-variable pump pulses at 1 μJ. The probe pulse was delayed relative to the pump pulse with a mechanical delay stage. Both beams were focused onto the sample in a pump–probe geometry, and interferograms were recorded by using a grating spectrometer (Princeton Instruments) coupled to a 64-pixel MCT array detector (Jackhammer, PhaseTech). A rotating frame, 4-frame phase cycling, and parallel polarization were used throughout the measurements. All data were collected with a waiting time of 300 fs. Samples for 2D IR measurements were prepared by lyophilizing the aggregation products and redissolving them in 20 μL of D_2_O. The sample solution was placed between two CaF_2_ windows separated by a 25 μm spacer.

## Supplementary Material



## Notes

Low-resolution cryo-EM processing of classes categorized as “short crossover” and fitting to the published model; MALDI-FTICR-MS analysis of coaggregation product washed with sarkosyl; aggregation kinetics of rIAPP seeded with hIAPP fibrils; representative cryo-EM 2D class averages for each collected data set and combined classification from 10,000 random particles of each data set; coaggregation kinetics similar to Figure 2 without inset; number of total picked particles and segments considered for final classification (PDF)

## Notes

Cryo-EM particles from this study are available from the corresponding author upon request.

## Notes

The peptides used in this study are human IAPP (UniProt: P10997), rat IAPP (UniProt: P12969).
