# Supplementary material for: Cryo-Electron Microscopy Provides Mechanistic Insights into Solution-Dependent Polymorphism and Cross-Aggregation Phenomena of the Human and Rat Islet Amyloid Polypeptides
Source: Biochemistry. 2025 May 26;64(12):2583–95. doi: 10.1021/acs.biochem.5c00042 (PMC12177924; doi:10.1021/acs.biochem.5c00042)
Supplement: Supplementary file 1 [file bi5c00042_si_001.pdf]

Supporting Information: Cryo-electron Microscopy Provides Mechanistic Insights into Solution-dependent Polymorphism and Cross-aggregation Phenomena of the human and rat Islet Amyloid Polypeptides

Dylan Valli,† Saik Ann Ooi,‡ Ibrahim Kaya,¶ Asger Berg Thomassen,§ Himanshu Chaudhary,† Tobias Weidner,§ Per E. Andrén,¶ and Michał Maj*,†

†*Department of Chemistry – Ångström Laboratory, Uppsala University, Lägerhyddsvägen 1, 751 20 Uppsala, Sweden*

‡*Department of Chemistry and Molecular Biology, University of Gothenburg, Medicinaregatan 7B, 413 90 Gothenburg, Sweden*

¶*Department of Pharmaceutical Biosciences, Spatial Mass Spectrometry, Science for Life Laboratory, Uppsala University, BMC 591, 75124 Uppsala, Sweden*

§*Department of Chemistry, Aarhus University, Langelandsgade 140, Aarhus C 8000, Denmark*

E-mail: michal.maj@kemi.uu.se

Supporting Information

1. Cryo-EM data processing of short crossover fibrils

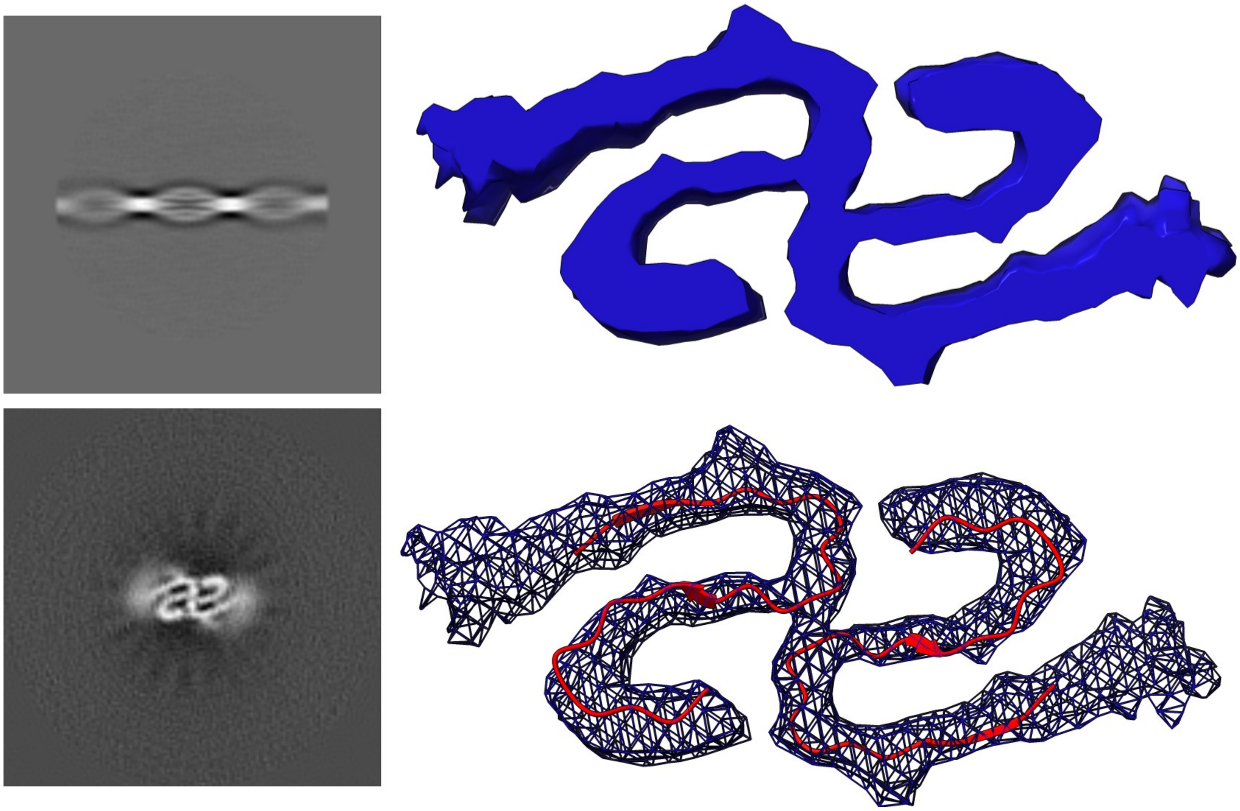

Figure S1: Low resolution Cryo-EM analysis of the polymorph corresponding to a crossover of 260 Å. In the top left, a representative of the 2D class averages used for further 3D classification. 3D classifications and refinement using a cylinder as initial model led to the post-processed map shown on the top right with a rise of X and twist of Y. Bottom left corresponds to a 3D slice obtained from the resulting map. The PDB entry 7M64 was fitted into this map with a high correlation on the bottom right, indicating that this structure corresponds to the same polymorph as the published ex vivo structure with the same helical parameters. Further higher resolution processing was not performed due to the low magnification used during data collection.

2. ThT–monitored kinetics of rIAPP seeded with hIAPP

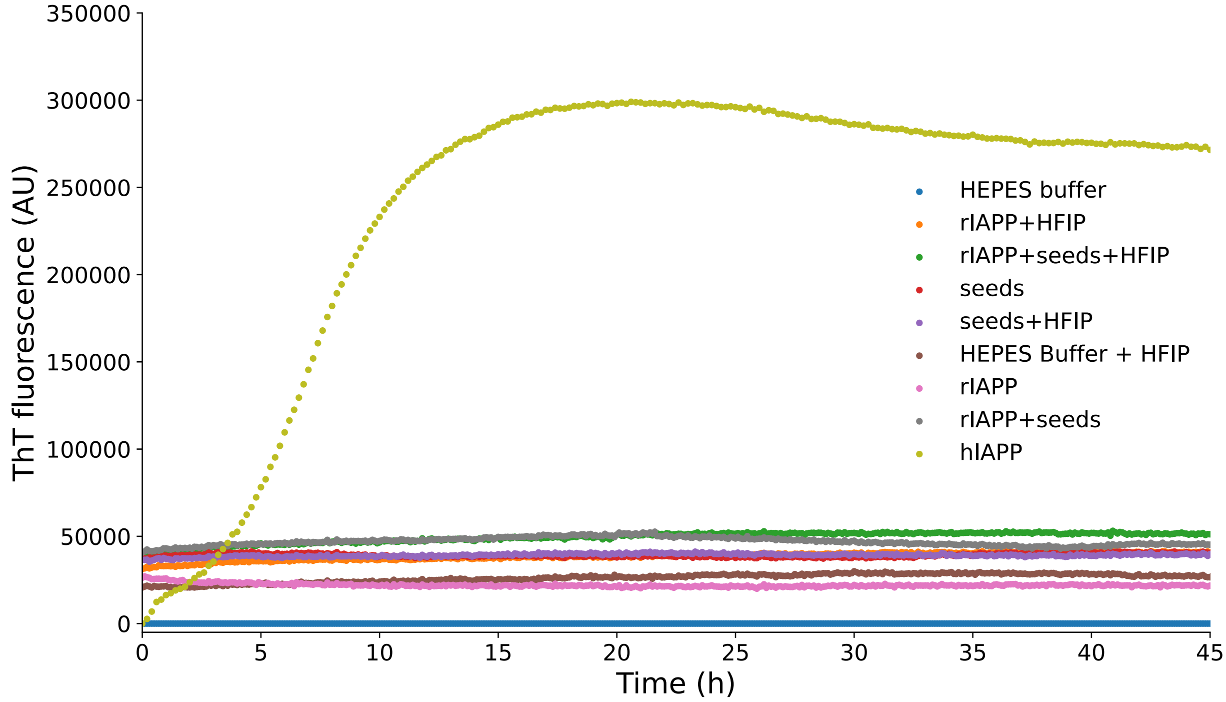

Figure S2: The aggregation of rIAPP seeded with hIAPP fibrils was monitored using ThT. hIAPP seeds were aggregated for 1 week at 100 µM in HEPES buffer. Seeds were sonicated and added to 100 µM of monomeric rIAPP at a ratio of 10% (v/v). hIAPP at 100 µM shows a typical aggregation curve while no aggregation is observed for the rIAPP samples with and without seeds.This suggests that coaggregation does not occur with preformed hIAPP fibrils but only when hIAPP is added as monomer.

3. Representative class averages for each dataset collected.

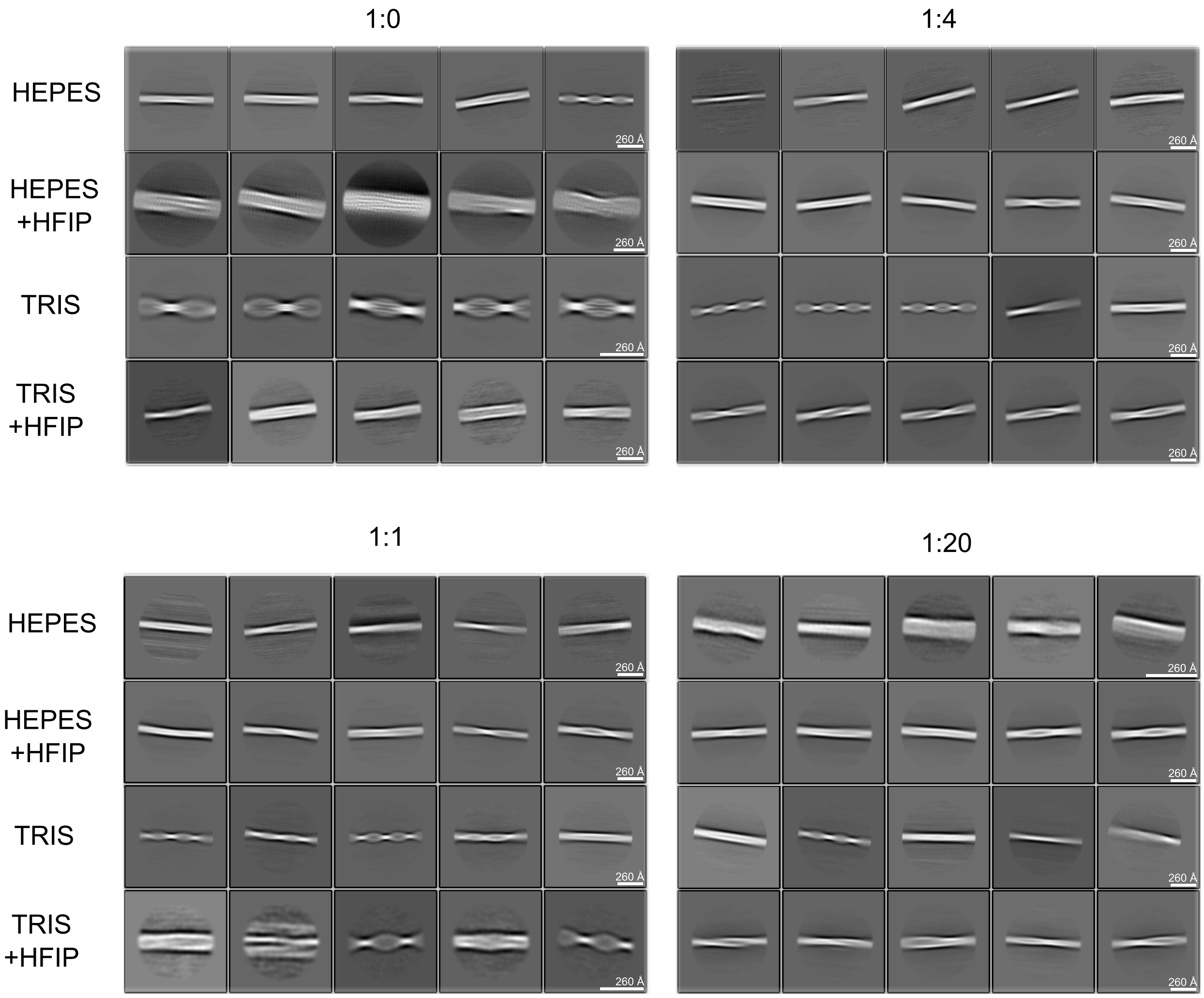

Figure S3: The five most populated class averages for each dataset after removal of non-fibrillar particles. All particles were initially extracted using the same box size. For some datasets, particles were re-extracted with a smaller box size and re-classified to improve assessment of fibril helicity.

4. MALDI-FTICR-MS analysis of the peptides present in fibrils after sarkosyl wash.

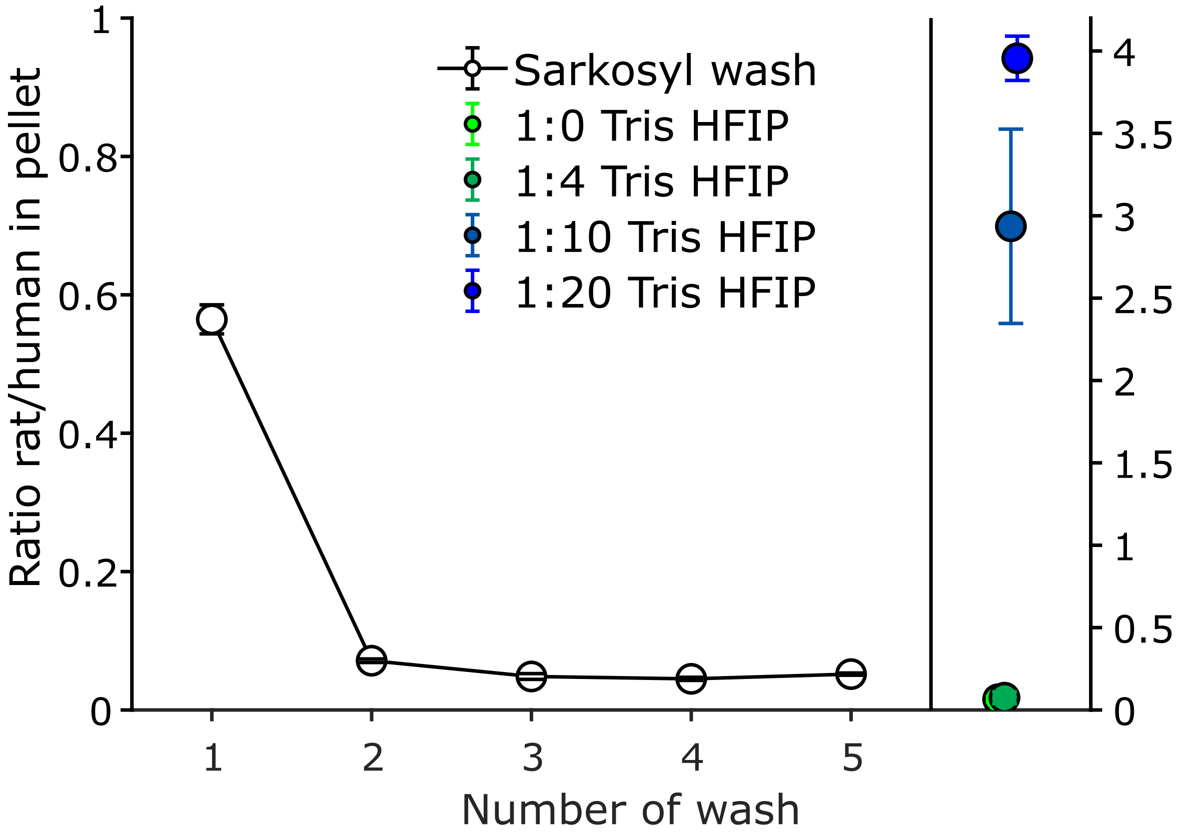

Figure S4: Matrix-assisted laser desorption/ionization (MALDI) Fourier-transform ion cyclotron resonance (FTICR) mass spectrometry analysis of fibrils formed by coaggregation of rat and human IAPP. The fibrils were washed with 1% sarkosyl to remove any peptide loosely associated with the fibril surface. In TRIS buffer (1:1 ratio), the rat IAPP signal largely disappears after three washes, suggesting surface-bound peptide removal. In contrast, when using TRIS HFIP buffer, the rat peptide signal remains high after 5 wash steps for higher initial rat concentrations. This indicates that the rat peptides are incorporated into the fibril core rather than simply coating the surface.
